# Two new species of the megadiverse lentic diving-beetle genus *Hydrovatus* (Coleoptera, Dytiscidae) described from NE Thailand

**DOI:** 10.3897/zookeys.632.10483

**Published:** 2016-11-16

**Authors:** Olof Biström, Johannes Bergsten

**Affiliations:** 1 Finnish Museum of Natural History, Zoology Unit, PB 17, FI-00014 University of Helsinki, Finland; 2 Swedish Museum of Natural History, Department of Zoology, Box 50007, SE-10405 Stockholm, Sweden

**Keywords:** Hydrovatus, Coleoptera, Dytiscidae, taxonomy, new species, keys to species, Thailand

## Abstract

Here we describe two new *Hydrovatus* species (Coleoptera: Dytiscidae: Hydroporinae: Hydrovatini) from the province of Khon Kaen, Isan region in NE Thailand. *Hydrovatus* is the third most species rich genus of diving beetles (Dytiscidae). It occurs on all continents except Antarctica and now numbers 210 currently recognized species. Both new species, *Hydrovatus diversipunctatus* **sp. n.** and *Hydrovatus globosus* **sp. n.**, were collected at lights and are only known from the type locality “Khon Kaen” (a city and province). Diagnoses based on morphology for the separation from closely related species are given together with illustrations of male genitalia and habitus photos. We provide a determination key to Old World species of the *pustulatus* species group and to Oriental species of the *oblongipennis* species group.

## Introduction

*Hydrovatus* in the subfamily Hydroporinae together with *Copelatus* (Copelatinae) and *Laccophilus* (Laccophilinae) are the only three genera of diving beetles (Dytiscidae) with more than 200 species (Nilsson 2016; Miller and Bergsten 2016). As genera they also share the characteristics of having a cosmopolitan distribution existing on all continents except Antarctica (Miller and Bergsten 2016). A good dispersal ability is probably part of this colonization success as witnessed by them all having common species that regularly come flying to lights (Miller and Bergsten 2016). Good dispersal ability in aquatic insects is commonly associated with inhabiting lentic waters (Ribera and Vogler 2000; Ribera et al. 2003; Hof et al. 2006; Hjalmarsson et al. 2015), and all three genera have lentic representatives. *Hydrovatus* however stands out in that lotic species are largely lacking (Balke 2005). This genus is truly characteristic of standing swamps and ponds rich in vegetation. It is surprising that a strictly lentic diving beetle genus has become megadiverse since the characteristic of good dispersal ability generally is linked with larger distribution ranges and, according to theory, a lower speciation rate (Ribera et al. 2001; Hjalmarsson et al. 2015; but see Letsch et al. 2016). Finally, while all three ‘megagenera’ may be considered difficult due to their diversity, *Hydrovatus* in contrast to *Copelatus* and *Laccophilus* have received a modern world monographic revision and is therefore more accessible on a global level (Biström 1997).

In terms of morphology *Hydrovatus* has a characteristic body shape with acuminate elytral apices and some males have modified antenna, both features rather uncommon in diving beetles. Further *Hydrovatus* have deeply incised metacoxal processes with long, slender metacoxal lobes and female gonocoxae are fused into a knife-like ovipositor (Miller and Bergsten 2016). As in the species-poor sister genus *Queda*, also in Hydrovatini, the apex of the prosternal process is broad and triangular (Miller and Bergsten 2014). Currently there are 208 species recognized in the genus (Nilsson 2016). Following the global monograph on the genus (Biström 1997), only a handful of new species have been described, mainly from western Africa (Bilardo and Rocchi 1999, 2008; Schizzerotto and Pederzani 2015), but also from the Oriental region (Biström 1999; Manivannan and Madani 2011).

Unsorted, unidentified, accession material in museum collections around the world are “gold mines” with likely tens of thousands of undescribed species waiting to be discovered (Balke et al. 2013). During a recent visit to Budapest in Hungary the senior author of this article had a chance to study the insect collection in the Hungarian Natural History Museum (HNHM). Among the unsorted diving beetles material in the collection two series of specimens from Khon Kaen in NE Thailand with peculiar body shapes were discovered. After examination under a dissection microscope both proved to belong to undescribed species, which we here describe. Referring to the revision of the genus *Hydrovatus* (Biström 1997) one of the new species belongs to the species group *pustulatus* (group 3) and the other, to the species group *oblongipennis* (group 11).

## Material and methods

The type material of both species is kept in the Hungarian Natural History Museum, Budapest, Hungary (HNHM), the Finnish Museum of Natural History, Helsinki, Finland (FMNH) and the Swedish Museum of Natural History, Stockholm, Sweden 
(NHRS). Habitus photographs were produced using a Canon EOS 5D Mark II DSLR camera with an MP-E 65mm f/2.8 1–5× macro lens mounted on a Stackshot (Cognisys) motorized rail. For light source the macro twin-head flash MT-24EX (Canon) was used with a home-made light diffusor. A Z-stack of 15–35 photos was taken by operating the Stackshot rail through the software Zerene stacker (Zerene Systems) and stacking the images in the same software to produce an image with focus throughout the globular body. Black and white line drawings of genitalia were produced using a Wild M11 dissection microscope with a camera lucida.

## Results

### Hydrovatus diversipunctatus sp. n.

Taxon classificationAnimaliaColeopteraDytiscidae

http://zoobank.org/112B3346-2DF9-49A3-8A7E-8CF204324ED6

#### Type locality.

Thailand: Khon-Kaen [city and province in the region of Isan, NE Thailand].

#### Type material

8 exs. (1 male, 7 females). Holotype, male: “Nordost-Thailand Khon-Kaen ad lucem / Dr. Sastri Saowakontha leg. 28.4.1980” (HNHM). - Paratypes: Same data as holotype (1 ex. FMNH); same data as holotype but “25.IV.1980” (1 ex. HNHM, 1 ex. NHRS); same data as holotype but “22.IV.1980” (2 exs. HNHM, 1 ex. FMNH); same data as holotype but “20.5.1980” (1 ex. HNHM).

#### Diagnosis.

**Figure 1.**
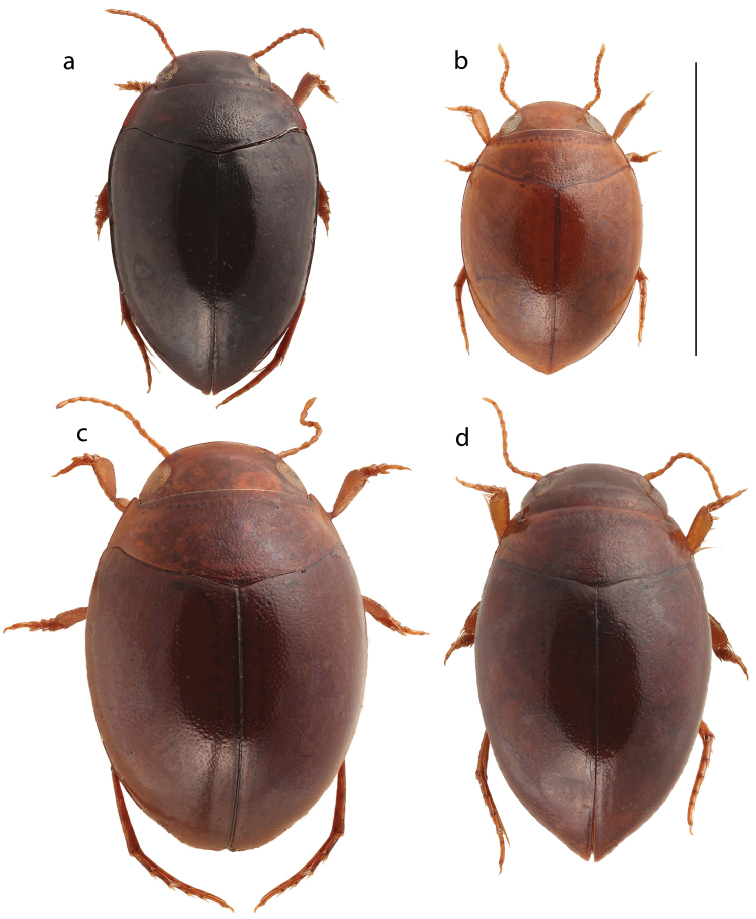
Dorsal habitus of *Hydrovatus diversipunctatus* sp. n. (**a**), *Hydrovatus subrotundatus* (**b**), *Hydrovatus globosus* sp. n. (**c**) and *Hydrovatus rufoniger rufoniger* (**d**). Scale bar 3 mm.

The new species is undoubtedly closest to *Hydrovatus subrotundatus* Motschulsky. These two species share the characteristics of having the lateral elytral margin clearly visible from above (Fig. 1a–b, compare with Fig. 1c–d). The two species are distinguished by clear difference in body size and shape; *Hydrovatus diversipunctatus* is larger and less rounded-globular than *Hydrovatus subrotundatus*. Moreover, *Hydrovatus diversipunctatus* deviates by having much coarser pronotal punctures in comparison with general punctation of elytra (diameter of pronotal punctures about 4× larger than general punctures of elytra). Additionally, head between eyes has complete frontal margin in *Hydrovatus subrotundatus*, while frontal margin in *Hydrovatus diversipunctatus* fades away close to eyes. Shape of male genitalia is quite similar in the two species. Penis is, however, slightly broader in *Hydrovatus diversipunctatus*, while parameres seem to be a little more slender, compared with corresponding structures in male genitalia of *Hydrovatus subrotundatus*.

#### Description.

Body: Almost entirely blackish ferrugineous, with no distinct color pattern. Body-shape not globular but slightly elongated. Broadest posterior to humeral region and from there posteriorly slightly narrowed until abruptly curved towards apex of elytra. Lateral margin between epipleura and elytra pronounced and clearly discernible from above (Fig. 1a). Length of body 3.1–3.3 mm, width 2.0–2.1 mm.

Head: Blackish ferrugineous; near frontal margin head slightly paler, dark ferrugineous. Very finely and sparsely punctate. At eyes and in rather shallow, frontal depressions with some fine punctures. Rather shiny, although finely microsculptured. Reticulation clearly discernible except on minor tubercles frontally close to eyes where reticulation is obliterated. Frontal outline of head rounded, medially slightly straightened. Frontal margin fades away on minor tubercles close to eyes. Antenna pale ferrugineous, slender and with no modifications.

Pronotum: Blackish ferrugineous, laterally with vague dark ferrugineous areas. Densely and distinctly punctate; laterally punctures become sparse and slightly finer. Rather shiny, although distinctly microsculptured; meshes clearly discernible. Sides of pronotum slightly rounded to almost straight; anteriorly distinctly curved inwards.

Elytra: Finely and sparsely punctate. Rows of punctures indistinct and weakly developed except from discal row, which basally is quite distinct. Rather shiny, although very finely microsculptured; reticulation weak but extensively still discernible. Narrowly, close to epipleura reticulation in part obliterated. Epipleura dark ferrugineous; finely punctate frontally at inner margin.

Ventral aspect: Dark ferrugineous, except abdomen, apically slightly paler, ferrugineous. Almost impunctate, except for metacoxal plates and metathorax, which in part are covered with fine to rather fine punctures. Rather shiny with fragments of microsculpture, except abdomen which is entirely microsculptured. Prosternal process laterally with fine margin; medial surface almost flat and punctured. No stridulatory apparatus on metacoxal plates.

Legs: Pale ferrugineous to ferrugineous. Pro- and mesotarsus slightly enlarged. Claws simple.

**Figure 2.**
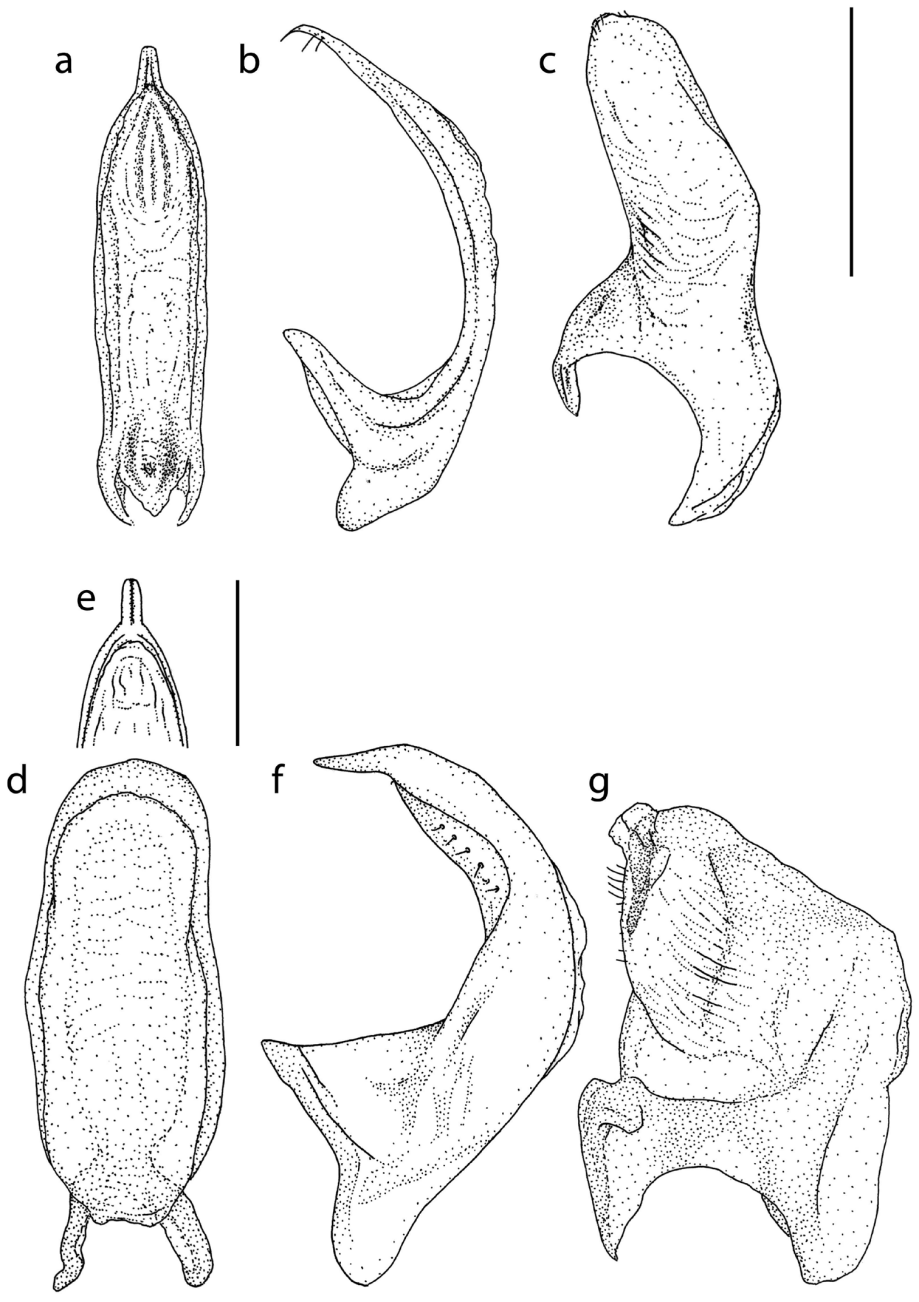
Male genitalia of *Hydrovatus diversipunctatus* sp. n. (**a–c**) and *Hydrovatus globosus* sp. n. (**d–g**). **a, d** penis, dorsal aspect **b, f** penis, lateral aspect **c, g** paramere **e** penis, frontal part from above. Scale bar in upper right corner 0.5 mm for **a–d, f–g**. Scale bar next to (**e**) 0.5 mm, applies to only **e**.

Male genitalia as in Fig. 2a–c.

Female: Externally similar to male.

#### Distribution.

Thailand.

#### Collecting circumstances.

Type material collected at light.

#### Etymology.

The species name *diversipunctatus* refers to the large difference in size between general punctures of pronotum in comparison to those of elytra.

### Hydrovatus globosus sp. n.

Taxon classificationAnimaliaColeopteraDytiscidae

http://zoobank.org/F8776495-71E1-4BEF-9AA2-365074523F52

#### Type locality.

Thailand: Khon-Kaen [city and province in the region of Isan, NE Thailand].

#### Type material

25 exs. (10 males, 15 females). Holotype, male: “Nordost-Thailand Khon Kaen, ad lucem 22.4. 1980 leg. S. Saowakontha” (HNHM). – Paratypes: Same data as holotype (11 exs. HNHM, 3 exs. FMNH, 3 exs. NHRS); same data as holotype but “20.5. 1980” (2 exs. HNHM, 1 ex. FMNH); same data as holotype but “29.4. 1980 Dr. Saati Saowakontha leg.” (1 ex. HNHM); same data as holotype but “2.9. 1980 Dr. Saati Saowakontha leg.” (1 ex. HNHM); same data as holotype but “19.2. 1981 Dr. Saati Saowakontha leg.” (2 exs. HNHM).

#### Diagnosis.

The new species belongs to a complicated group of *Hydrovatus*, out of which *Hydrovatus rufoniger* (Clark) (Fig. 1d) seems to be closest. The new species is distinguished from this species but also other close species from the Oriental region, by its globular shaped body with a very weak extension of the elytral apex (Fig. 1c). Deviating structures in the shape of the penis apex are also characteristic for *Hydrovatus globosus* (Fig. 2d–f).

#### Description.

Body: Almost unicolored ferrugineous to dark ferrugineous; no distinct color-pattern exhibited. Body-shape almost globular with apex of elytra moderately extended (Fig. 1c). Length 3.8–4.0 mm, width 2.4–2.6 mm.

Head: Anteriorly between eyes finely margined; outline slightly undulate (frontal edge medially, weakly curved inwards). At each eye with a quite distinct, triangular depression with irregular punctures in it. Close to eye with a row of fine punctures and from frontal depression a row of punctures continues sparsely (disappears gradually) towards middle of head. Other parts of head surface impunctate with scattered, fine and hardly discernible punctures anteriorly. Head slightly matte to rather shiny; extensively finely reticulated. Antenna filiform, with no distinct modifications.

Pronotum: With dense and fine punctures, which laterally fade away gradually. Surface between punctures shiny, almost without reticulation. Laterally, fine, in part indistinct reticulation discernible.

Elytra: Finely and densely punctate. Laterally, punctures fade away and become indistinct/disappear in part. Discal, dorsolateral and lateral row of punctures rather indistinct and in part hardly discernible. Between punctures, surface rather shiny; reticulation very fine and sporadically discernible; extensively reticulation almost absent.

Ventral aspect: Finely to fairly finely and somewhat sparsely punctate. Abdomen almost impunctate. Shiny, reticulation almost absent; hardly visible, rudimentary meshes of microsculpture discernible on metacoxal plates. Abdomen slightly matte; with very fine, elongated meshes of microsculpture. Stridulation apparatus rather narrow, provided with numerous minute striae. Apex of prosternal process laterally finely margined; medial surface flattened with sparse and vague punctures. Apical ventrite medially with a distinct depression; extreme apex of ventrite with a fine bulb (a minor enlargement).

Legs: Ferrugineous. Pro- and mesotarsus slightly enlarged. Protarsal claws asymmetric; internal claw distinctly angled and thickened.

Male genitalia as in Fig. 2d–g.

Female: Elytra posteriorly rather distinctly microsculptured, matte. Protarsal claws not modified. No stridulation apparatus on metacoxal plates.

#### Distribution.

Thailand.

#### Collecting circumstances.

Entire type material collected at light.

#### Etymology.

The species name *globosus* refers to the spherical body-shape of the new species.

## Determination keys

For comparisons, see illustrations in Biström (1997).

Key to Old World species of the *pustulatus* species group (sp. gr. 3 sensu Biström 1997):

**Table:** 

1	Lateral margin between elytron and epipleuron for a long distance not discernible from above (as in Fig. 1a–b)	**2**
–	Lateral margin between elytron and epipleuron discernible from above (Fig. 1c–b)	**3**
2	Elytra provided with distinct, pale ferrugineous spots; penis (lateral aspect) slender	***Hydrovatus cardoni* Severin, 1890**
–	Elytra provided with narrow, marginal, pale ferrugineous spots; penis (lateral aspect) broad	***Hydrovatus sringeriensis* Manivannan & Madani, 2011**
3	Smaller species (length of body 2.3–2.9 mm), rufotestaceous and rather compact (Fig. 1b); no clear difference in size of punctures on pronotum and elytra; penis (dorsal aspect) not expanded	***Hydrovatus subrotundatus* Motschulsky, 1859**
–	Larger species (length of body 3.1–3.3 mm), darker ferrugineous and more elongate (Fig. 1a); punctures on pronotum distinctly larger than on elytra (punctures hardly visible); penis (dorsal aspect) slightly expanded	***Hydrovatus diversipunctatus* sp. n.**

Key to Oriental species of the *oblongipennis* species group (sp. gr. 11 sensu Biström 1997). The taxonomic status of *Hydrovatus castaneus*, *Hydrovatus rufoniger* and *Hydrovatus bonvouloiri* is unclear and in need of further study (synonymies cannot be excluded):

**Table:** 

1	Small species, length of body 2.2–2.7 mm	***Hydrovatus seminarius* Motschulsky, 1859**
–	Larger species, length of body 3.0–4.2 mm	**2**
2	Metacoxal plates (males) lack stridulation apparatus	***Hydrovatus rufescens* Motschulsky, 1859**
–	Metacoxal plates (males) with stridulation apparatus	**3**
3	Body shape globular; apical extension of elytra indistinct (Fig. 1c)	***Hydrovatus globosus* sp. n.**
–	Body shape elongated; apex of elytra distinct, posteriorly clearly extended (Fig. 1d)	**4**
4	Penis apex (dorsal aspect) narrows smoothly to tip	***Hydrovatus castaneus* Motschulsky, 1855**
–	Penis apex (dorsal aspect) narrows abruptly/unevenly to tip	**5**
5	Penis apex broad, narrows abruptly to slender tip; ridges of stridulatory file larger, clearly discernible; male protarsal claws not distinctly thickened	***Hydrovatus picipennis* Motschulsky, 1859**
–	Penis apex more slender and narrows less abruptly to slender tip; ridges of stridulatory file very fine, hardly discernible; male protarsal claws distinctly thickened	**6**
6	Penis apex (lateral aspect) with protruding frontal flaps	***Hydrovatus naviger* Biström, 1997**
–	Penis apex (lateral aspect) lacks frontal flaps	**7**
7	Penis (dorsal aspect) medially broad, narrows evenly forwards to slender tip; elytral punctures fine to rather fine (Fig. 1d)	***Hydrovatus rufoniger* (Clark, 1863)**
–	Penis (dorsal aspect) medially broad, narrows more abruptly forwards to slender tip; elytral punctures sometimes coarser	***Hydrovatus bonvouloiri* Sharp, 1882**

## Supplementary Material

XML Treatment for Hydrovatus diversipunctatus

XML Treatment for Hydrovatus globosus
